# Gelsolin Promotes Cancer Progression by Regulating Epithelial-Mesenchymal Transition in Hepatocellular Carcinoma and Correlates with a Poor Prognosis

**DOI:** 10.1155/2020/1980368

**Published:** 2020-01-08

**Authors:** Yixi Zhang, Xiaojing Luo, Jianwei Lin, Shunjun Fu, Pei Feng, Hongjun Su, Xiangjun He, Xue Liang, Kunpeng Liu, Wen Deng

**Affiliations:** ^1^Organ Transplant Center, First Affiliated Hospital, Sun Yat-sen University, Guangzhou 510080, China; ^2^Department of Medical Oncology, Sun Yat-sen University Cancer Center, Sun Yat-sen University, Guangzhou, China; ^3^Organ Transplant Center, Shenzhen Third People's Hospital, Shenzhen, China; ^4^Department of Liver Surgery, First Affiliated Hospital, Sun Yat-sen University, Guangzhou 510080, China; ^5^Department of Hepatobiliary Surgery, Zhujiang Hospital, Southern Medical University, Guangzhou 510280, China; ^6^Guangdong Provincial Key Laboratory of Liver Disease, Cell-Gene Therapy Translational Medicine Research Center, The Third Affiliated Hospital of Sun Yat-sen University, Guangzhou 510630, China; ^7^Department of Nutrition, School of Public Health, Sun Yat-Sen University, Guangzhou, China; ^8^Biotherapy Department, Sun Yat-sen Memorial Hospital, Sun Yat-sen University, 107 Yan Jiang West Road, Guangzhou, China

## Abstract

Gelsolin (GSN), a cytoskeletal protein, is frequently overexpressed in different cancers and promotes cell motility. The biological function of GSN in hepatocellular carcinoma (HCC) and its mechanism remain unclear. The expression of GSN was assessed in a cohort of 188 HCC patients. The effects of GSN on the migration and invasion of tumour cells were examined. Then, the role of GSN in tumour growth *in vivo* was determined by using a cancer metastasis assay. The possible mechanism by which GSN promotes HCC progression was explored. As a result, GSN was overexpressed in HCC tissues. High GSN expression was significantly correlated with late Edmondson grade, encapsulation, and multiple tumours. Patients with high GSN expression had worse overall survival (OS) and disease-free survival (DFS) than those with low GSN expression. GSN expression was identified as an independent risk factor in both OS (hazard risk (HR) = 1.620, 95% confidence interval (CI) = 1.105–2.373, *P* < 0.001) and DFS (HR = 1.744, 95% CI = 1.205–2.523, *P*=0.003). Moreover, GSN knockdown significantly inhibited the migration and invasion of HCC tumour cells, while GSN overexpression attenuated these effects by regulating epithelial-mesenchymal transition (EMT) In conclusion, GSN promotes cancer progression and is associated with a poor prognosis in HCC patients. GSN promotes HCC progression by regulating EMT.

## 1. Introduction

Hepatocellular carcinoma (HCC) is one of the most common cancers and the fourth leading cause of cancer-related death worldwide [1]. Seventy percent of HCC patients suffer from tumour recurrence. Metastasis contributes predominantly to recurrence, which causes the high mortality of HCC patients [2]. However, the molecular mechanisms of HCC metastasis remain unclear. Therefore, understanding the potential mechanisms of HCC development is an important first step towards the discovery of novel effective treatments.

Cancer dissemination is largely associated with cell migration and invasion. Gelsolin (GSN) is an actin-binding protein and a key regulator of actin filament assembly and disassembly that promotes cell motility. It is located in the cytoplasm, mitochondria, and plasma [3]. GSN was reported to be associated with the lamellipodia formation of endothelial cells [4]. However, the correlation between GSN expression and metastasis remains controversial. GSN was initially recognized as a tumour suppressor and is downregulated in certain types of cancer, including human breast, colorectal, gastric, bladder, and non-small-cell lung cancers [5–9]. Conversely, several reports have found that GSN is upregulated in a subset of pancreatic and gynaecologic cancers and acts as an oncogene [10, 11].

It has been reported that GSN is upregulated in both human HCC tissue and the hepatocellular cell line Hca-F, which has high lymphogenous metastatic potential [12, 13]. A few studies have revealed that GSN is associated with the process of epithelial-mesenchymal transition (EMT) in breast and gastric cancers [8, 14, 15]. However, the mechanisms by which GSN mediates the metastasis of HCC are not well defined.

To further elucidate the role of GSN in HCC, we investigated GSN expression in human HCC and adjacent noncancerous tissues and the effect of GSN on invasion and migration in HCC cells. We found that GSN expression was higher in HCC tissues than in adjacent liver tissues, whereas GSN expression was higher in metastatic HCC tumour tissues than in nonmetastatic HCC tumour tissues. Furthermore, our results showed that GSN promotes HCC cell migration and invasion *in vitro*, and the knockdown of GSN attenuates HCC metastasis *in vivo*, potentially by influencing the EMT process.

To further elucidate the role that GSN plays in HCC, we investigated GSN expression in HCC, its impact on patient survival, and its relationship with the tumour stage. We also studied the molecular mechanisms by which GSN modulates cancer. Our results revealed that GSN promotes the migration and invasion of HCC cells *in vitro*, and the knockdown of GSN attenuates HCC metastasis *in vivo* by influencing the EMT process.

## 2. Methods

### 2.1. HCC Patient Data and Tissue Specimens

A total of 188 HCC tissue specimens were obtained from patients undergoing hepatectomy at the First Affiliated Hospital, Sun Yat-sen University (Guangzhou, China) between January 2006 and December 2008. Another 40 pairs of matched HCC and adjacent noncancerous liver tissue samples were obtained during the operation between January 2009 and May 2013. The samples were snap frozen in liquid nitrogen and stored in a −80°C freezer for subsequent experiments, such as RNA extraction and formalin fixation and paraffin-embedded immunohistochemistry. Disease-free survival (DFS) and overall survival (OS) were used to assess efficacy. The follow-up of the patients was performed as described in our previous work [16]. The deadline for follow-up was 31 October 2015. We collected the patients' complete clinical and pathological features. The study followed the Ethical Guidelines of the 1975 Helsinki Declaration, which were revised in 2013. All patients received informed consent on the use of their clinical specimens for medical research. The use of human body materials was approved by the Ethics Committee of the First Affiliated Hospital of Sun Yat-sen University.

### 2.2. Cell Lines and Cell Culture

The human HCC cell line HepG2 was purchased from the American Type Culture Collection (ATCC; Rockville, MD, USA). The human HCC cell lines HepG3B, HCCLM3, SMMC-7721, and Bel7402 and the normal hepatocyte cell line L02 were all obtained from the Institute of Biochemistry and Cell Biology, Chinese Academy of Sciences and verified.

Cell lines were cultured in low-glucose Dulbecco's Modified Eagle Medium (DMEM) containing 10% foetal bovine serum (FBS), 100 U/ml penicillin, and 0.1 mg/ml streptomycin at 37°C and 5% CO_2_.

### 2.3. Immunohistochemical Analysis of Gelsolin

Immunohistochemistry (IHC) was performed using Gelsolin antibodies (G4896, Sigma) as described in our previous work [17]. Immunohistochemical analysis was performed by two independent researchers who were blinded to patient data. Shimizu criteria [18] were used to score the expression of Gelsolin protein in HCC specimens from 0 to 3+. Patients with a score of 0 or 1+ were defined as the low expression group, while those with a score of score of 2 or 3+ were defined as the high expression group.

### 2.4. Producing High- and Low-GSN-Expressing HCC Cell Lines

We purchased a GSN overexpression plasmid from Forevergen Biosciences (Guangzhou, China). The sequence of the GSN-specific antisense oligonucleotide is 5′-UUCAGAACAAAGGCAUCGdTdT-3′. The sequence of the control oligonucleotide is 5′-UUCCGAACGUGUCACGUdTdT-3′. HCCLM3 cells were transfected with a GSN small hairpin RNA (shRNA) or a scramble shRNA, and as previously described, SMMC-7721 cells were transfected with the GSN overexpression plasmid or a vector control [19]. Next, 0.5 mg/mL purinomycin was used to select the transfected cells. Quantitative real-time polymerase chain reaction (qRT-PCR) and western blot (WB) analysis were used to confirm the stably transfected clones.

### 2.5. qRT-PCR and WB Analysis

qRT-PCR and WB analysis were performed as previously described [20]. qRT-PCR was used to analyse the transcripts of *β*-actin (control) and GSN. The primers were purchased from Invitrogen (Thermo Fisher Scientific, Inc.). The primer sequences were as follows: *β-actin*, forward 5′-AGCGAGCATCCCCCAAAGTT-3′ and reverse 5′-GGGCACGAAGGCTCATCATT-3′ and *GSN*, forward 5′-GGTGTGGCATCAGGATTCAAG-3′ and reverse 5′-TTTCATACCGATTGCTGTTGGA-3′.

### 2.6. Transwell Migration and Invasion Test

For the transwell migration assay, serum-free DMEM containing 5 × 10^4^ cells was inoculated into the upper chamber of 8 *μ*m transwell inserts (BD Biosciences, Franklin Lakes, NJ), and DMEM containing 10% bovine serum albumin (BSA) was added to the lower chamber. After incubation at 37°C for 24 hours, the cells in the upper chamber were carefully removed, and the cells attached to the lower side of the transwell membrane were fixed in 20% methanol and stained with 0.1% crystal violet. An inverted microscope (Nikon, Chiyoda-Ku, Japan) was used to examine the number of cells. For the transwell invasion assay, all procedures were the same as those described for the transwell migration assay, except that the upper chamber was coated with Matrigel (BD Biosciences, Franklin Lakes, NJ).

### 2.7. *In Vivo* Lung Metastasis Model

*In vivo* metastasis determination was performed in male BALB/c thymus-free nude mice (3-4 weeks old) obtained from the Animal Center of Guangdong Province (Guangzhou, China). The mice were randomized and treated in a nonblinded manner. The use of laboratory animals was based on guidelines from the National Institutes of Health. For the lung metastasis model, HCC cells (1 × 10^6^) suspended in 200 *μ*L of phosphate-buffered saline (PBS) were injected intravenously through the tail vein (5 mice in each group). At the point of termination, the mice were euthanized. Lungs were excised, and metastasis was confirmed by haematoxylin-eosin (HE) staining. The animal experiments were approved by the Life Animal Use Committee of the First Affiliated Hospital of Sun Yat-sen University.

### 2.8. Statistical Analysis

SPSS software (19.0; SPSS, Inc., Chicago, IL) was used for statistical analysis. The measurement data are expressed as the mean ± standard deviation (SD). *Student's t-test* was used for intergroup comparisons. Categorical data were analysed by the *chi-square test* or Fisher's exact test. Kaplan–Meier's method and the logarithmic rank test were used to analyse the survival rate. A Cox regression model of multivariate competition risk was used to further analyse the factors identified as significant (*P* < 0.1) in the univariate analysis to identify significant independent predictors of DFS and OS in HCC patients. A forward step-by-step programme was used for variable selection and the final multivariable model. *P* < 0.05 was considered statistically significant.

## 3. Results

### 3.1. GSN Is Overexpressed in HCC Tissues

**Figure 1:**
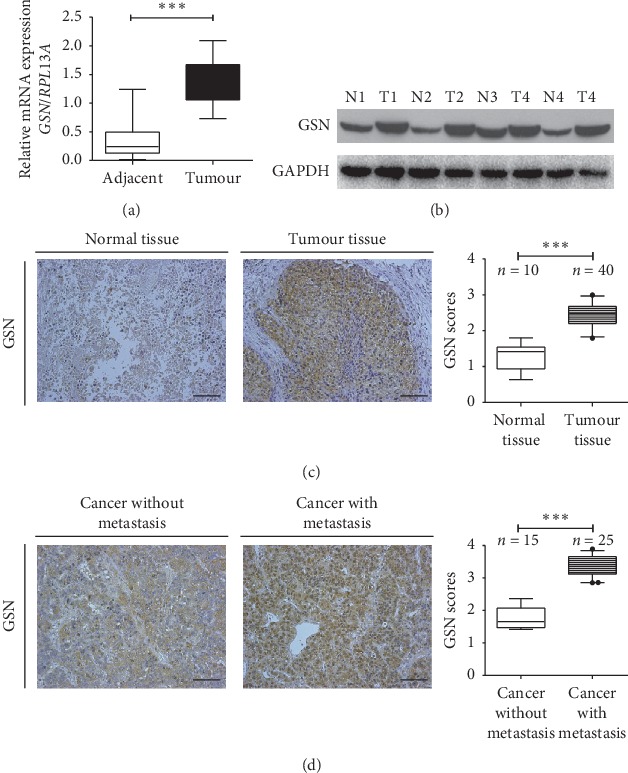
Gelsolin is upregulated in hepatocellular carcinoma. Gelsolin expression measured by quantitative PCR (a) and western blot (b) and immunohistochemistry (c, 100x) analysis in hepatocellular carcinoma (HCC) tissues compared with adjacent normal tissues. Gelsolin expression measured by immunohistochemistry in metastatic HCC compared with nonmetastatic HCC (d, 200x). ^*∗∗∗*^*P* < 0.001 (*Student's t-test*).

To investigate the potential clinical relevance of GSN and HCC progression, GSN mRNA and protein levels in paired HCC specimens and adjacent noncancerous liver tissues were measured. The levels of both GSN mRNA and protein were significantly upregulated in HCC tissues compared with adjacent normal liver tissues (Figures 1(a) and 1(b)). Immunohistochemical analysis indicated that GSN mainly localizes within the cytoplasm of HCC tissues, in accord with previous reports, and the intensity of GSN-positive staining was markedly increased in HCC tissues compared with that in noncancerous tissues (Figure 1(c)). We also evaluated the difference in GSN expression between metastatic HCC tissues and HCC tissues *in situ* (Figure 1(d)). Because GSN expression is higher in HCC tissues, the DAB color development time of the IHC was 20 minutes as in Figure 1(c). In order to show the difference between metastatic and nonmetastatic tissues, in the IHC assay of Figure 1(d), we used a shorter IHC chromogenic time (5 minutes), so the IHC results in the nonmetastatic group were lower and the staining was shallow.

### 3.2. High GSN Expression Is Associated with a Poor Prognosis in HCC Patients

We assessed whether GSN expression was associated with clinicopathological factors in patients with HCC. The 188 patients with HCC were divided into two groups according to the IHC results: the GSN high expression group (*n* = 95) and the GSN low expression group (*n* = 93). The results showed that high GSN expression in HCC was positively correlated with an advanced Edmondson grade (*P*=0.022), capsulation (*P*=0.006), and multiple tumour lesions (*P*=0.012); however, high GSN expression in HCC was not significantly associated with age, gender, a family history of HCC, alpha-fetoprotein (AFP), cirrhosis, or tumour size (*P* > 0.05) (Table 1).

**Figure 2:**
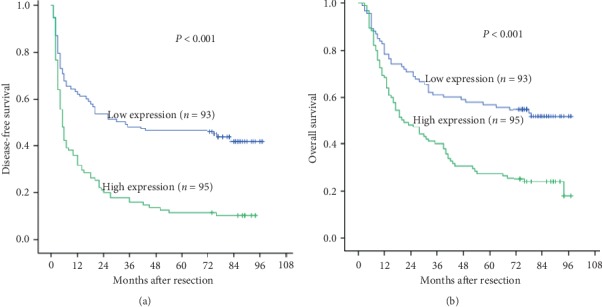
GSN expression is associated with poor outcome of human HCC patients. Kaplan–Meier survival curves of DFS and OS for the GSN low expression group (*n* = 93) and the GSN high expression group (*n* = 95) based on the results of immunohistochemistry. The results show that HCC patients with low GSN expression have better DFS (a) and OS (b) than those with high expression of GSN.

Furthermore, we explored the prognostic value of GSN expression. We found that the 3- and 5-year DFS rates (15.8% and 11.6% vs. 48.4% and 46.2%, *P* < 0.001) and OS rates (40.0% and 27.4% vs. 61.3% and 57.0%, *P* < 0.001) of HCC patients in the high GSN expression group were worse than those in the low GSN expression group (Figures 2(a) and 2(b)). Kaplan–Meier analysis showed that AFP, Edmondson grade, tumour size, envelope, tumour number, vascular invasion, GSN expression, and tumour-node-metastasis (TNM) stage were risk factors for DFS and OS (Table 2). From the multivariate Cox regression analysis, high GSN expression was found to be an independent prognostic factor, with low DFS (hazard risk (HR) = 1.744, 95% confidence interval [CI]: 1.205–2.523, *P*=0.003) and OS (HR) = 1.620, 95% CI: 1.105–2.373, *P* < 0.001) (Table 3). These results indicate that high GSN expression is significantly associated with a poor prognosis, suggesting a potential role for GSN in liver tumourigenesis.

### 3.3. GSN Is Associated with HCC Migration and Invasion

**Figure 3:**
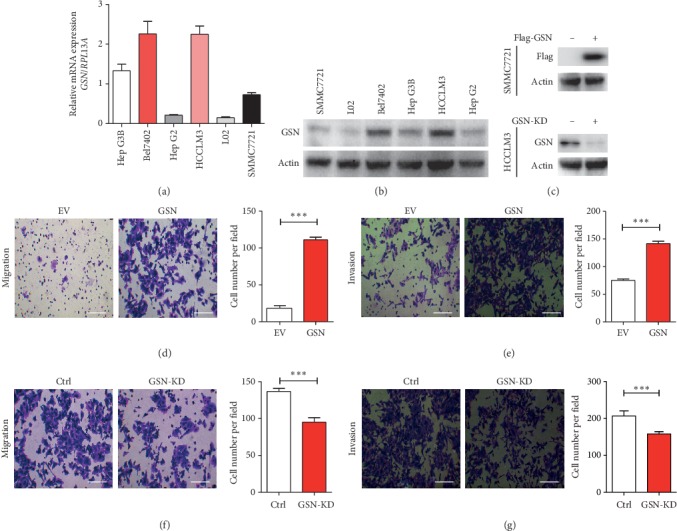
Gelsolin is associated with HCC migration and invasion. Quantitative-PCR (a) and western blot analysis (b) of GSN expression levels in a panel of HCC cell lines. GSN plasmid overexpressed GSN in SMMC7221 cells compared with control vector and GSN shRNA inhibited the expression of GSN in HCCLM3 cells compared with scramble shRNA (c). Overexpression of GSN enhances migration (d) and invasion (e) in SMMC7221 cells. Knockdown of GSN with shRNA inhibits migration (f) and invasion (g) in HCCLM3 cells. ^*∗∗∗*^*P* < 0.001 (Student's *t*-test).

To examine GSN expression in HCC cells, qRT-PCR and WB analysis were performed in five hepatoma cell lines, namely, SMMC7721, HepG2, HCCLM3, HepG3B, and Bel7402 and the liver cell line L02 as a normal control. The mRNA levels of GSN in HepG2 and HCCLM3 cells were higher than those in the L02 cell line, while the GSN levels in the other three cell lines were lower (Figure 3(a)). The protein expression levels of GSN were consistent with the mRNA levels (Figure 3(b)).

GSN is frequently reported to be critical for cell migration and invasion [8, 19, 21, 22]. Considering the correlation between GSN expression and metastasis, we performed an immunohistochemistry assay to investigate GSN as a potential modulator of cell motility in HCC. To determine how GSN affects the motility of HCC cells, we overexpressed GSN in SMMC7721 cells and knocked down GSN in HCCLM3 cells (Figure 3(c)). Then, we measured the migration and invasion abilities of these two new cell lines. Compared with vector-transfected cells, the relative number of migrating and invading SMMC7721 cells transfected with GSN was significantly higher (Figures 3(d) and 3(e)). In contrast, the knockdown of GSN resulted in deceased migration and invasion in HCCLM3 cells (Figures 3(f) and 3(g)). These results suggest that GSN functions as a positive modulator of HCC metastasis that promotes the migration and invasion of HCC cells.

### 3.4. GSN Promotes EMT in HCC Cells

**Figure 4:**
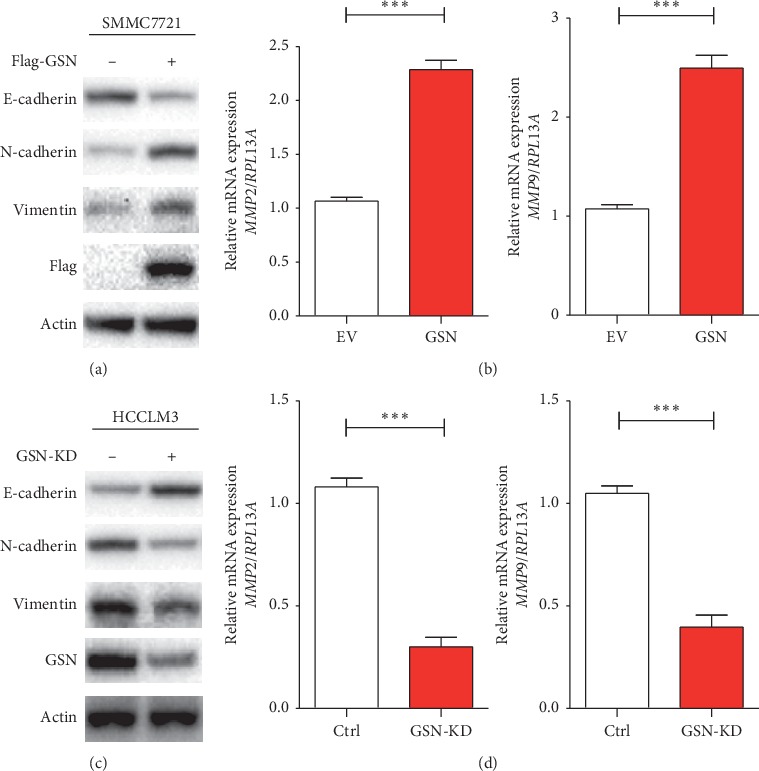
GSN promotes EMT of HCC cells. Western blot analysis of markers associated with EMT. Overexpression of Gelsolin by plasmid showed reduced levels of E-cadherin and enhanced levels of N-cadherin and vimentin in SMMC7221 cells (a). Quantitative PCR shows increased levels of MMP2 and MMP9 (b). Knockdown of GSN with shRNA shows increased levels of E-cadherin and reduced levels of N-cadherin and vimentin in HCCLM3 cells (c). Quantitative PCR shows decreased levels of MMP2 and MMP9 (d). ^*∗∗∗*^*P* < 0.001 (Student's t-test).

Tumour metastasis, which involves the migration and invasion of tumour cells, is always associated with EMT. We next wanted to determine whether GSN affects HCC cell motility by modulating EMT and performed western blot analysis after GSN overexpression and GSN knockdown. In GSN-overexpressing SMMC7721 cells, we observed the downregulation of E-cadherin, an epithelial marker, and the upregulation of N-cadherin, a mesenchymal marker. Vimentin was also upregulated (Figure 4(a)). In contrast, E-cadherin was increased, and N-cadherin was decreased in GSN-knockdown HCCLM3 cells. Vimentin was downregulated (Figure 4(c)). Matrix metalloproteinase (MMP)2 and MMP9, which are known as zinc-dependent endopeptidases, are tightly associated with tumourigenesis and metastasis and are essential for mesenchymal cell invasion. We found that MMP2 and MMP9 mRNAs were significantly upregulated in SMMC7721 cells with GSN overexpression and downregulated in HCCLM3 cells with GSN knockdown (Figures 4(b) and 4(d)). These results show that GSN promotes EMT in HCC cells, thereby enhancing cell migration and invasion.

### 3.5. Knockdown of GSN Attenuates HCC Metastasis *In Vivo*

**Figure 5:**
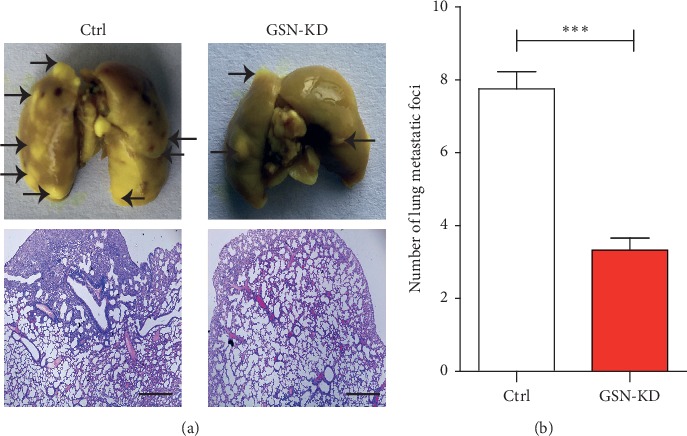
Knockdown of GSN attenuates HCC metastasis *in vivo*. HCCLM3 cells transfected with GSN shRNA or scramble shRNA were inoculated through the tail vain into nude Balb/c mice. Mice were sacrificed at the endpoint. Lungs were harvested. Metastatic foci were confirmed by gross observation and further by HE staining (a). The number of metastatic nodules was calculated (b). ^*∗∗∗*^*P* < 0.001 (Student's t-test).

Furthermore, to explore the biological importance of GSN in HCC, we examined the growth of tumours in xenotransplantation experiments. Human tumour cells were injected into nude mice by tail vein injection, and the growth of tumours was monitored. After 8 weeks, the incident rate and the number of pulmonary metastatic nodules decreased significantly in the sh-GSN group compared with the sh-NC group. Representative histomorphological gross and HE staining images are shown in Figures 5(a) and 5(b). These results indicate that GSN knockdown effectively reduces the metastatic lung tumour burden. Targeting GSN could effectively decrease HCC metastasis *in vivo*.

## 4. Discussion

Cancer metastasis is extremely complex and can be influenced by thousands of factors. Aberrant cell motility and dysregulation of the tumour microenvironment are both important causes of cancer metastasis [23]. The migration and invasion of tumour cells across the tissue barrier require the degradation of specific components of the extracellular matrix. GSN was initially reported to be reduced in many cancers, including gastric, breast, and colorectal cancers, and is recognized as a tumour suppressor gene [6–8, 24]. However, it has been reported that GSN upregulates and promotes cell migration and invasion [10, 11]. Previous studies have also shown that GSN is involved in the regulation of tumour metastasis [8, 15], but the association is unclear. The role of GSN in different types of cancer is controversial.

In this study, we first detected the expression of GSN in 188 HCC tissues and 5 cell lines and found that GSN was upregulated in both tissues and cells. Second, we explored whether GSN expression is associated with the prognosis of HCC patients after surgery. After further experiments, we confirmed that GSN could be an independent factor in the postoperative prognosis in patients with HCC. These results suggest that GSN may play an important role in HCC progression. Therefore, we studied how GSN promotes the progression of HCC.

Tumour invasion is often associated with the loss of epithelial markers and the acquisition of mesenchymal markers, including migration and motile behaviour, referred to as EMT. The concept of EMT provides a new way to identify genes that are important for the progression of cancer to a dedifferentiated and more malignant state. Increasing evidence suggested that transforming growth factor-beta 1 (TGF-*β*1) triggered epithelial to mesenchymal transition (EMT). Chen et al. [14] discovered that, in the TGF-*β*1-enriched cells, the mRNA expression levels for the markers of mesenchymal cell (i.e., N-cadherin and vimentin) were found to be increased in concomitance with the increased expression for GSN. This study indicated that GSN may involve in the EMT process of tumour cells. We found that GSN plays an inhibitory role in HCC metastasis by modulating EMT. We first measured the migration and invasion abilities of GSN-overexpressing or GSN-knockdown HCC cells and revealed that GSN promotes cell migration and invasion. *In vivo*, we established a murine lung metastasis model of HCC and found that the knockdown of GSN significantly reduced the number of lung metastatic nodules. Mechanistically, we knockeddown GSN and discovered upregulated E-cadherin and downregulated N-cadherin and vimentin. In previous studies, MMP2 and MMP9 were identified to play a role in promoting and enhancing metastasis [24]. We found a reduction in MMP2 and MMP9 mRNAs, whereas GSN overexpression produced the opposite results. However, the specific mechanism by which GSN interacts with and affects EMT remains to be determined.

Among the metastasis of solid tumour, vascular invasion is an important part of *de novo* tumour growth. It is known that vascular invasion can be extensively stimulated by the target gene of the EMT-signalling pathway, such as MMP9. MMP9 produced by MDSCs modulates tumour vascularization, hence promotes tumour progression and metastasis [25, 26]. Besides, it is reported that elevated GSN expression is associated with angiogenesis in an *in vitro* model of VEGF-A-induced angiogenesis [27], indicating that GSN may promote vascularization in tumour tissue. Our work revealed that GSN can promote EMT signalling and subsequent tumour invasion. These evidence provide hints for that GSN may promote vascular invasion of HCC cells.

In this study, our findings suggest that the overexpression of GSN is associated with an aggressive tumour phenotype and a poor prognosis in HCC patients after hepatectomy. In addition, *in vitro* and *in vivo* measurements confirmed the role of GSN in promoting HCC progression. Furthermore, we demonstrated that GSN enhances HCC cell motility and facilitates metastasis by regulating EMT. Therefore, we propose that strategies designed to downregulate GSN in HCC patients with high GSN expression might provide a promising way to mitigate the progression of HCC. In summary, we determined the high expression of GSN in HCC tissues and also indicated the oncogenic roles of GSN in HCC which might provide a novel strategy for targeted therapy of HCC overexpressing GSN.

## Figures and Tables

**Table 1:** Comparisons of clinicopathological features of HCC patients with GSN.

Category	Subcategory	Cases	GSN		*P* value
			Low expression	High expression	
Gender	Male	166	79	87	0.157
	Female	22	14	8	

Age (years)	≤50	100	49	51	0.891
	>50	88	44	44	

HCC family history	Yes	18	8	10	0.654
	No	170	85	85	

HBsAg	Negative	28	19	9	0.035
	Positive	160	74	86	

AFP (ng/ml)	<200	83	45	38	0.247
	≥200	105	48	57	

Edmonson grading	I-II	142	77	65	0.022
	III-IV	46	16	30	

Tumour size (cm)	≤5	69	37	32	0.386
	>5	119	56	63	

Liver cirrhosis	Absent	39	16	23	0.236
	Present	149	77	72	

Capsulation	Capsulated	130	73	57	0.006
	Noncaspulated	58	20	38	

Tumour number	Single	136	75	61	0.012
	Multiple	52	18	34	

Vascular invasion	Yes	33	13	20	0.202
	No	155	80	75	

HBsAg, hepatitis B surface antigen; AFP, alpha fetoprotein.

**Table 2:** Prognostic factors for DFS and OS by univariate analysis.

Variables	*n*	DFS			OS		
		3 yrs	5 yrs	*P*	3 yrs	5 yrs	*P*
Gender							
Male	166	30.7%	27.1%		49.4%	40.4%	
Female	22	40.9%	40.9%	0.195	59.1%	54.5%	0.112

Age (years)							
≤50	100	32.0%	27.0%		48.0%	39.0%	
>50	88	34.1%	30.7%	0.282	53.4%	45.5%	0.334

HCC family history							
Yes	18	27.8%	16.7%		55.6%	55.6%	
No	170	32.9%	30.0%	0.451	50.0%	40.6%	0.226

HBsAg							
Negative	28	46.4%	46.4%		67.9%	53.6%	
Positive	160	29.4%	25.6%	0.030	47.5%	40.0%	0.073

AFP (ng/ml)							
<200	83	43.4%	42.2%		62.7%	54.2%	
≥200	105	22.9%	18.1%	<0.001	41.0%	32.4%	0.003

Edmondson grading							
I-II	142	37.3%	34.5%		55.6%	48.6%	
III-IV	46	15.2%	10.9%	<0.001	34.8%	21.7%	0.001

Tumour size (cm)							
≤5	69	53.6%	47.8%		73.9%	65.2%	
>5	119	19.3%	17.6%	<0.001	37.0%	28.6%	<0.001

Liver cirrhosis							
Absent	39	30.8%	25.6%		59.0%	46.2%	
Present	149	32.2%	29.5%	0.771	48.3%	40.9%	0.186

Capsulation							
Capsulated	130	40.0%	37.7%		60.8%	51.5%	
Noncaspulated	58	13.3%	8.6%	<0.001	27.6%	20.7%	<0.001

Tumour number							
Single	136	40.4%	36.0%		61.0%	52.9%	
Multiple	52	9.6%	9.6%	<0.001	23.1%	13.5%	<0.001

Vascular invasion							
Yes	33	3.0%	3.0%		15.2%	6.1%	
No	155	38.1%	34.2%	<0.001	58.1%	49.7%	<0.001

GSN							
Low expression	93	48.4%	46.2%		61.3%	57.0%	
High expression	95	15.8%	11.6%	<0.001	40.0%	27.4%	<0.001

DFS, disease-free survival; OS, overall survival; other abbreviations are as in Table 1.

**Table 3:** Prognostic factors for DFS and OS by the multivariate Cox proportional hazards regression model.

Variables	DFS			OS		
	HR	95% CI	*P*	HR	95% CI	*P*
AFP	1.980	1.380–2.842	<0.001	1.697	1.156–2.490	0.007
Edmondson grading	1.480	1.014–2.162	0.042			
Tumour size	1.942	1.313–2.871	0.001	1.944	1.252–3.020	0.003
Capsulation	0.601	0.410–0.881	0.009	0.514	0.339–0.779	0.002
Tumour number	0.588	0.404–0.854	0.005	0.494	0.330–0.738	0.001
Vascular invasion	2.022	1.271–3.216	0.003	1.957	1.213–3.156	0.006
GSN	1.744	1.205–2.523	0.003	1.620	1.105–2.373	0.013

## Data Availability

The data used and analyzed in this study are available from the corresponding author upon request.
